# A Missing Connection: A Review of the Macrostructural Anatomy and Tractography of the Acoustic Radiation

**DOI:** 10.3389/fnana.2019.00027

**Published:** 2019-03-07

**Authors:** Chiara Maffei, Silvio Sarubbo, Jorge Jovicich

**Affiliations:** ^1^Harvard Medical School, Athinoula A. Martinos Center for Biomedical Imaging, Massachusetts General Hospital, Charlestown, MA, United States; ^2^Center for Mind/Brain Sciences - CIMeC, University of Trento, Trento, Italy; ^3^Division of Neurosurgery, Structural and Functional Connectivity Lab Project, S. Chiara Hospital, Trento Azienda Provinciale per i Servizi Sanitari (APSS), Trento, Italy; ^4^Department of Psychology and Cognitive Sciences, University of Trento, Trento, Italy

**Keywords:** acoustic radiation, auditory system, sensory pathways, auditory pathways, auditory tract, diffusion-based tractography

## Abstract

The auditory system of mammals is dedicated to encoding, elaborating and transporting acoustic information from the auditory nerve to the auditory cortex. The acoustic radiation (AR) constitutes the thalamo-cortical projection of this system, conveying the auditory signals from the medial geniculate nucleus (MGN) of the thalamus to the transverse temporal gyrus on the superior temporal lobe. While representing one of the major sensory pathways of the primate brain, the currently available anatomical information of this white matter bundle is quite limited in humans, thus constituting a notable omission in clinical and general studies on auditory processing and language perception. Tracing procedures in humans have restricted applications, and the *in vivo* reconstruction of this bundle using diffusion tractography techniques remains challenging. Hence, a more accurate and reliable reconstruction of the AR is necessary for understanding the neurobiological substrates supporting audition and language processing mechanisms in both health and disease. This review aims to unite available information on the macroscopic anatomy and topography of the AR in humans and non-human primates. Particular attention is brought to the anatomical characteristics that make this bundle difficult to reconstruct using non-invasive techniques, such as diffusion-based tractography. Open questions in the field and possible future research directions are discussed.

## Introduction

The acoustic radiation (AR) represents a highly-myelinated group of axonal projections and constitutes one of the primary sensory pathways of the primate brain, carrying auditory information from the thalamus to the cortex. The connectivity pattern of these fibers has been described in some detail in cytoarchitectonic and myeloarchitectonic studies of non-human primates (Polyak, 1932; Mesulam and Pandya, 1973; Morel et al., 1993; Hackett et al., 1998) and, at a more macroscopic level, in a few histological studies in humans (Flechsig, 1920; Pfeifer, 1920; Rademacher et al., 2002; Bürgel et al., 2006). However, the information obtained from non-human primate studies cannot be transferred directly to the human brain. Furthermore, such studies have focused mostly on the cytoarchitectonic aspects of the auditory cortices and their intrinsic connectivity, with little emphasis on the anatomical course of the AR itself. In humans, tracing studies are impossible *in vivo* and have restricted applications in post-mortem brains (Mesulam, 1979; Tardif and Clarke, 2001), while limited information can be drawn from old myeloarchitectonical post-mortem studies.

The advent of diffusion magnetic resonance imaging (dMRI) (Basser et al., 1994) and tractography (Mori et al., 1999) has made it possible to investigate the anatomy of the major white matter (WM) bundles of the human brain *in vivo* and non-invasively (Catani et al., 2002; Catani and de Schotten, 2008; Lawes et al., 2008). However, the AR constitutes a notable exception in this sense. This primary sensory bundle is largely absent from most tractography studies investigating audition and language and from human WM atlases (Thiebaut de Schotten et al., 2011). This is mainly due to the intrinsic anatomical characteristics of these fibers, which go beyond the current limits of dMRI tractography methods (Behrens et al., 2007; Jones and Cercignani, 2010; Daducci et al., 2016). Therefore, the diffusion-based tractography reconstruction of the AR remains highly challenging at present, discouraging its *in vivo* anatomical investigation in humans.

However, overcoming or circumventing these methodological considerations is essential, as successful *in vivo* reconstruction of the human auditory tract is of great importance for both clinical applications (e.g., pre-surgical mapping) and basic neurobiological research. Reliably revealing the 3D characteristics of this tract would help in correlating the anatomical and functional aspects of audition and in the study of human-only cognitive functions, such as language, both in healthy and pathological conditions.

The main aim of this review is to emphasize the paramount need for characterizing the human AR in both clinical and scientific contexts, which has yet to be done for a number of reasons that we discuss. This review collates the available information from primate studies on the anatomy, topography, and course of the AR, with particular emphasis on the anatomical features that make this tract extremely challenging to study, even for state-of-the-art dMRI tractography techniques. Additionally, recent attempts to reconstruct the AR using diffusion-based tractography methods will be discussed. Finally, open questions in the field will be presented and possible future research directions considered.

## The Acoustic Radiation in Primates

**FIGURE 1:**
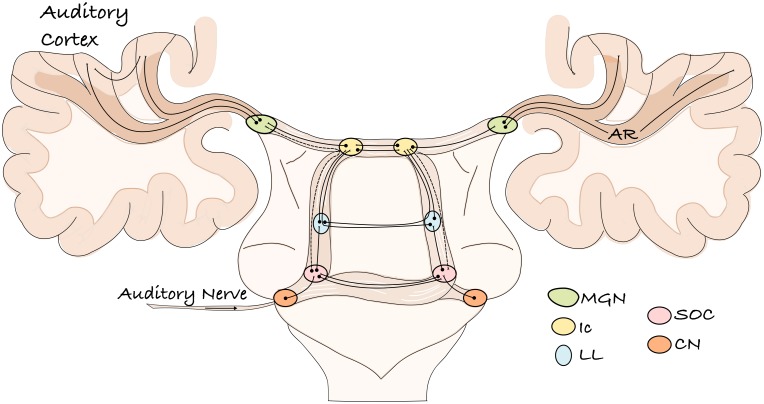
A schematic representation of the ascending human auditory system. The auditory information enters through the auditory nerve and reaches the cochlear nucleus. From here, various projections transmit the information to different brainstem relays of both hemispheres and then to the auditory cortex. The lemniscal pathway is represented by solid lines; the extra-lemniscal pathway by dashed lines. CN, cochlear nucleus; SOC, superior olivary complex; LL, lateral lemniscus; Ic, inferior culliculus; MGN, medial geniculate nucleus; AR, acoustic radiation.

The auditory system of mammals is a complex network of parallel and overlapping axonal projections that connect subcortical nuclei and cortical regions. It encodes and transmits stimuli coming from the acoustic environment, enabling an organism to detect a sound in its environment, determine the direction from which it originated, discriminate among potential sources, and thereby, react or communicate with conspecifics. Figure 1 provides a schematic representation of the human ascending auditory system and its sub-cortical relays, as it is commonly described in both the scientific literature and neuroanatomy textbooks (Moller, 2006; Brugge, 2013). The thalamo-cortical projections of this system, which connects the medial geniculate nucleus (MGN) to auditory cortex, constitute the AR (Figure 1).

Anatomical knowledge of the human AR mainly comes from pioneering investigations at the beginning of the 20th century (Dejerine and Dejerine-Klumpke, 1895; Flechsig, 1920; Pfeifer, 1920). Since these early studies, very limited additional information on this structure has been reported for humans (Rademacher et al., 2001, 2002; Bürgel et al., 2006). Most of the anatomical and functional organization of the mammalian auditory system has been inferred from animal studies, mainly non-human primates and cats (Morel and Kaas, 1992; Hashikawa et al., 1995; Hackett et al., 1998; Kaas and Hackett, 2000; Hackett et al., 2001; Jones, 2003; de la Mothe et al., 2006; Lee and Winer, 2008). However, these studies primarily focus on the topographical mapping between the thalamus and auditory cortex without documenting the spatial connection pattern and course of these fibers (Jones, 2003; Hackett, 2011). As a consequence, most neuroanatomical books only report schematic drawings of this pathway (e.g., Figure 1). Furthermore, although comparative studies have shown similar features across human and non-human primate brains, cortical and subcortical architectonic differences, as well as cognitive dissimilarities, exist (Galaburda and Sanides, 1980; Galaburda and Pandya, 1983; Schmahmann et al., 2007; Passingham, 2009; Thiebaut de Schotten et al., 2012). Thus, it is important to understand the commonalities among primate species and to identify potentially unique aspects of the human auditory system that might be related to our ability to perceive and process language-specific stimuli. *In vivo* diffusion imaging techniques constitute a powerful tool in investigating these topics.

In the next section, we briefly review the AR microstructural topography, as described in animal studies, and its macrostructural anatomy, which has been gleaned from human research. Our goal is to provide a more complete anatomical profile of this bundle across species and to highlight the existing gap of topographical information between invasive and non-invasive studies. In particular, we focus on the AR *in vivo* imaging literature, reviewing recent attempts to visualize this tract using tractography techniques.

### The Acoustic Radiation in Invasive Studies

The axonal connections between the thalamus and the auditory cortex have been investigated in animals using different invasive techniques. These fibers stem from the medial geniculate nucleus of the thalamus (MGN), as first described by von Monakow (1882), and contact a specific area on the posterior part of the Sylvian fissure (Minkowski, 1923; Polyak, 1932), which has been described as a “rudimentary transverse temporal gyrus” (Walker, 1937) in monkeys. In most species, three major divisions of the MGN are identified: ventral (or principal), dorsal (or posterior), and medial (or magnocellular) (Winer et al., 2001; Jones, 2003). Each of these divisions has unique connections to different cortical regions. The ventral division receives input from the central nucleus of the inferior culliculus (Ic) and almost exclusively projects to what is defined as the core region of the auditory cortex (Mesulam and Pandya, 1973; Burton and Jones, 1976; Morel and Kaas, 1992; Morel et al., 1993; Hashikawa et al., 1995; Rauschecker et al., 1997). This region is distinguished by dense immunoreactivity for the calcium-binding protein parvalbumin, as most of its inputs come from the ventral MGN parvalbumin immunoreactive cells (Molinari et al., 1995), even if some connections with the other MGN divisions appear to exist (Luethke et al., 1989; Morel et al., 1993). This region occupies a portion of the caudal superior temporal plane (postero-medial part of the Heschl’s gyrus in humans) and it is characterized by a dense population of small granule cells (e.g., koniocortex) with a well-developed layer IV (Merzenich and Brugge, 1973; Seldon, 1981). Both the ventral MGN and the core region show a tonotopical organization in which the representation of frequencies is spatially organized. This suggests a topographical organization of fibers connecting similar frequency domains in these two structures (Burton and Jones, 1976; Molinari et al., 1995). The core region is surrounded by a secondary narrow belt region and a third, more lateral region that occupies the lateral surface of the superior temporal gyrus (Hackett et al., 1998; Kaas and Hackett, 2000). This latter “para-belt” region is generally considered to be a higher-order auditory region or auditory association cortex that integrates auditory with non-auditory multisensory information (Hackett et al., 1998). These regions are less responsive to pure tone sounds, preferring more complex sounds, and do not show the clear tonotopical organization typical of the core region (Rauschecker et al., 1995; Jones, 2003). The dorsal and medial divisions of the MGN constitute the major inputs to these secondary auditory association regions. These nuclei receive inputs from the external nucleus of the Ic, as well as from lower brainstem relays, and bypass the core region to project to secondary auditory and other cortical regions (Rauschecker et al., 1997; Jones, 2003; Winer and Lee, 2007).

Projections from the ventral MGN to the core region correspond to the most direct classic auditory pathway, also called the lemniscal pathway. These parallel connections are tonotopically organized and their neurons show sharp responses to tones (Morel et al., 1993). Direct projections from the other MGN divisions to secondary auditory cortical regions are part of the extralemniscal (or non-lemniscal) auditory pathway. These fibers are separate from but lie adjacent to those of the lemniscal pathway in the ascending auditory system. In addition, their subdivision continues at the cortical level, where the non-lemniscal pathway has stronger and more diffuse connections with regions surrounding the core region (Lee and Sherman, 2010). These pathways are less tonotopically organized and their neurons demonstrate fewer sharp responses to sounds.

**FIGURE 2:**
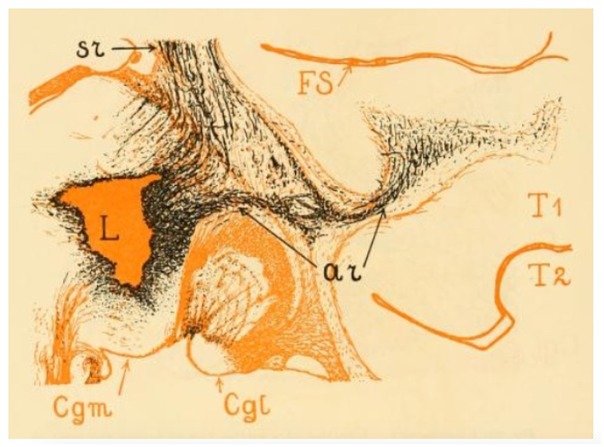
The image shows the acoustic radiation fibers in the rhesus monkey. The lesion (L) was located in the posterior thalamus. From here we can see numerous thalamocortical (or somato sensory) (sr) and auditory (ar) fibers emerging. The sr and the ar fibers form a system of which the ar occupies the most ventral position. The acoustic radiation occupies the upper half of the white matter of the superior temporal convolution (T1) and enters the cortex of the lower wall of the Sylvian fissure (FS). The level of this figure is immediately behind the posterior extremity of the lentiform nucleus; the entire length of the acoustic radiation is visible here. Cgm, medial geniculate nucleus; Cgl, lateral geniculate nucleus (adapted from Polyak, 1932; https://archive.org, public domain).

Classical studies in non-human primates focus on the topography of the connectivity between the MGN and the auditory projection cortical territory but provide no information on either the course and extension of the AR tract itself or its relationship with the other WM pathways of the brain. Using Marchi axonal degeneration, Polyak (1932) provided a detailed description of the course of this tract in rhesus macaques (Figure 2). He describes a dense bundle of closely assembled fibers that leaves the MGN, turns laterally and crosses the most ventral portion of the internal capsule (IC) immediately above the lateral geniculate nucleus (LGN) of the thalamus (Figure 2). At this level, these fibers are distinguishable from somato-sensory fibers because of their nearly horizontal orientation. The AR then bends ventrally and reaches the external capsule (EC) by passing through the ventral edge of the posterior putamen. Once there it meets other projection and association bundles before finally reaching the WM of the superior temporal convolution close to the Sylvian fissure (Figure 2). He describes the AR as a regularly arranged projection system, where fibers lie parallel to one another until gradually diverging only when approaching the cortex.

**FIGURE 3:**
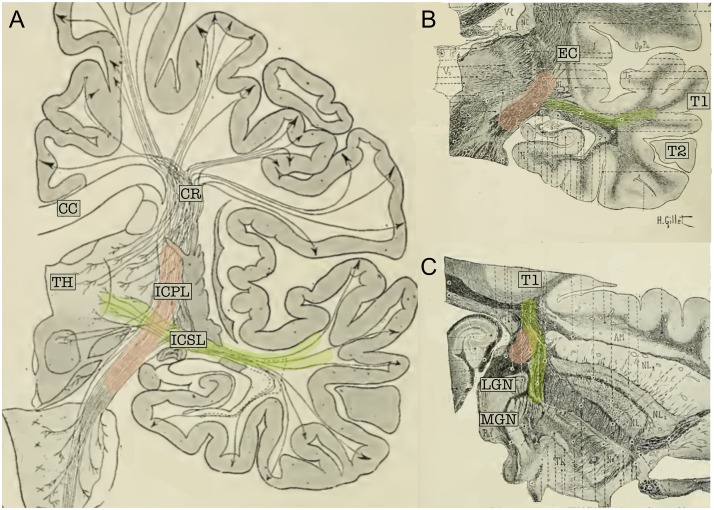
**(A)** Schematic representation of the projection fibers of the human brain (coronal view). **(B)** Coronal cut through the middle section of the thalamus. **(C)** Axial section of the brain; cut through the inferior thalamus. In the three panels, fibers belonging to the acoustic radiation have been highlighted in green and fibers of the internal capsule in pink. The acoustic radiation projects from the thalamus (TH) to the first temporal circonvolution (T1) and passes through the sub-lenticular and posterior segment of the internal capsule. This map clearly highlights the crossing between these two fiber systems. CC, Corpus callosum; EC, external capsule; ICPL, Posterior limb of the internal capsule; ICSL, sub-lenticular part of the internal capsule; CR, corona radiata; LGN, lateral geniculate nucleus; MGN, medial geniculate nucleus; T1, first temporal circonvolution; TH, thalamus (adapted from Dejerine and Dejerine-Klumpke, 1895; https://archive.org, public domain).

Classical topographical descriptions in humans (Figure 3) provide a very similar description (Dejerine and Dejerine-Klumpke, 1895; Flechsig, 1920; Pfeifer, 1920) of the AR, which may be summarized as follows. The AR leaves the MGN and travels in an antero-lateral direction; it then passes through the posterior portion of the IC, proceeds along the corona radiata and curves around the inferior portion of the circular sulcus of the insula before entering the transverse temporal gyrus of Heschl (HG) in a ventral-to-dorsal direction (Pfeifer, 1920). At this macrostructural level, both animal and human studies delineate a bundle with a transverse orientation that lies adjacent to and crosses over other main WM bundles before reaching the auditory cortex. In both animals and humans, the AR intermingles with the fibers of the IC in its most posterior portion. Furthermore, Dejerine and Dejerine-Klumpke (1895) described the close proximity of the AR to the optic radiation (OR) at the stemming point in the thalamus, defining this region as the “*carrefour sensitive*” (sensory intersection). Polyak (1932) also studied this region, stating that the OR and AR, although lying close together, are completely separate: the AR is located more anterior and crosses at a right angle above the OR, which follows a posterior direction in the sagittal plane. As will be discussed in the following section, this configuration and certain other anatomical features of the AR pose serious challenges to its 3D tractography reconstruction.

The myeloarchitectonic maps from Dejerine and Dejerine-Klumpke (1895) and Flechsig (1920), while being of invaluable historical significance, cannot be used to extract precise anatomical information that can be applied to modern brain atlases or neuroimaging studies. More recently, radiological information about the AR anatomical organization was obtained in human post mortem myelin-stained sections (Rademacher et al., 2002; Bürgel et al., 2006). These studies confirm the classical topographical description of the acoustic fibers and are of great importance as they represent the main reference framework for *in vivo* imaging studies of this brain region and provide the opportunity to investigate inter-subject variability and hemispheric asymmetry. Previous studies have found that the AR does not enter the lenticular nucleus, but rather runs dorsally to the OR, crossing the temporal isthmus as it ascends to the auditory cortex (Pfeifer, 1920; Polyak, 1932; Bürgel et al., 2006). Fanning of these fibers in HG creates a hat-like structure that covers the posterior end of the lenticular nucleus (Rademacher et al., 2002).

According to Flechsig (1920), these fibers are divisible into two bundles, one of which ascends near the Ec and enters the auditory cortex from the superior-posterior side. The other bundle courses for some distance in the company of the OR before passing behind and below the *fossa sylvii* where it pierces the bases of the middle and inferior temporal gyri to reach the transverse temporal gyrus or gyri. Similarly, early evidence from animal studies suggests that the AR is subdivided into dorsal and ventral components (von Monakow, 1882), although, Rademacher et al. (2002) found more recently only a single and heavily myelinated bundle.

Overall, the macrostructural description of the AR in humans resembles the description of this tract in non-human primates. The origin and termination of this bundle, together with the extension and relationship to other WM bundles, is maintained. However, the macro- and micro-anatomical correspondence between the different cortical regions in monkeys and humans is not straightforward (Baumann et al., 2013). Compared to non-human primates, the human cortical surface of the auditory regions demonstrates additional gyri and higher inter-subject and interhemispheric variability (Galaburda et al., 1978; Hackett et al., 2001), both of which may affect the AR anatomy. The human auditory cortex also shows higher differentiation into sub-regions as compared to non-human primates, and the core region is larger than the belt region (the opposite is true for monkeys) (Fullerton and Pandya, 2007). Both the differences in cortical anatomy between non-human primates and humans and the major variability of cortical and subcortical structures across subjects and hemispheres in humans (Bürgel et al., 2006) raise interesting questions about the possibility of a relationship between such morphological differences and human-only language abilities.

### The Acoustic Radiation in Non-invasive Tractography Studies

Diffusion MRI (dMRI) tractography allows for the investigation of WM architecture in the human brain non-invasively *in vivo*. Since its first applications (Mori et al., 1999), most of the well-known WM bundles of the human brain have been reconstructed using diffusion-based tractography methods (Catani and Thiebaut de Schotten, 2008; Lawes et al., 2008). Despite its potentials, dMRI tractography has several important limitations that have been discussed in depth in the literature (e.g., Jones and Cercignani, 2010; Thomas et al., 2014; Maier-Hein et al., 2017). In principle, these limitations affect most tractography reconstructions, but here we focus on how they particularly affect the 3D reconstruction of the AR. Reconstructing the AR three-dimensionally is highly challenging at present due to the anatomical features described in the previous sections: its relatively small size, transversal orientation, and location in a region with a high density of crossing fibers. This largely prevents the inclusion of this particular tract in most tractography investigations.

As shown in the previous section (see Figure 2, 3), in its medio-lateral course from the MGN to the HG, the AR lies in a nearly horizontal position and, for this reason, crosses some of the major fiber systems of the human brain: internal capsule, external capsule, and posterior thalamic radiation (Maffei et al., 2018). Resolving the fiber crossing is a well-known challenge in dMRI (Tuch et al., 2002; Dell’Acqua and Catani, 2012; Jeurissen et al., 2013). The classic tensor model (Basser et al., 1994) is capable of characterizing only one main fiber orientation per voxel and it has been shown to constantly fail in regions where voxels contain complex fiber architectures (Behrens et al., 2007; Jbabdi and Johansen-Berg, 2011). The impact of this limitation is particularly evident for non-dominant tracts, given that the orientation produced by the tensor will be closest to the largest contributing direction in most cases. This effect is amplified at the low resolution of commonly available diffusion protocols due to within-voxel partial volume averaging effects (Tournier et al., 2011). When implemented in tractography studies, the diffusion tensor model has proven to be incapable of detecting the 3D profile of the AR. Streamlines are either truncated when entering voxels containing major inferior-superior orientations or erroneously embedded in the reconstruction of these major projection bundles, with no visible streamlines contacting the HG (Behrens et al., 2007; Crippa et al., 2010; Berman et al., 2013). This has likely been the primary factor preventing the investigation of the auditory system by means of diffusion-based tractography. Some studies have used the diffusion tensor to investigate the WM microstructure of the auditory system, limiting the structural investigation of the auditory pathways to the extraction of mean quantitative diffusion measures [e.g., fractional anisotropy (FA)] from specific regions of interest (ROI) (Chang et al., 2004; Lee et al., 2007; Lin et al., 2008; Wu et al., 2009). However ROI-based analysis can lead to inaccurate results, especially for WM tracts that are extremely variable across subjects, such as the AR (Rademacher et al., 2002). Therefore, it is typically preferable to map the exact anatomy of such WM tracts in individual subjects/patients.

**FIGURE 4:**
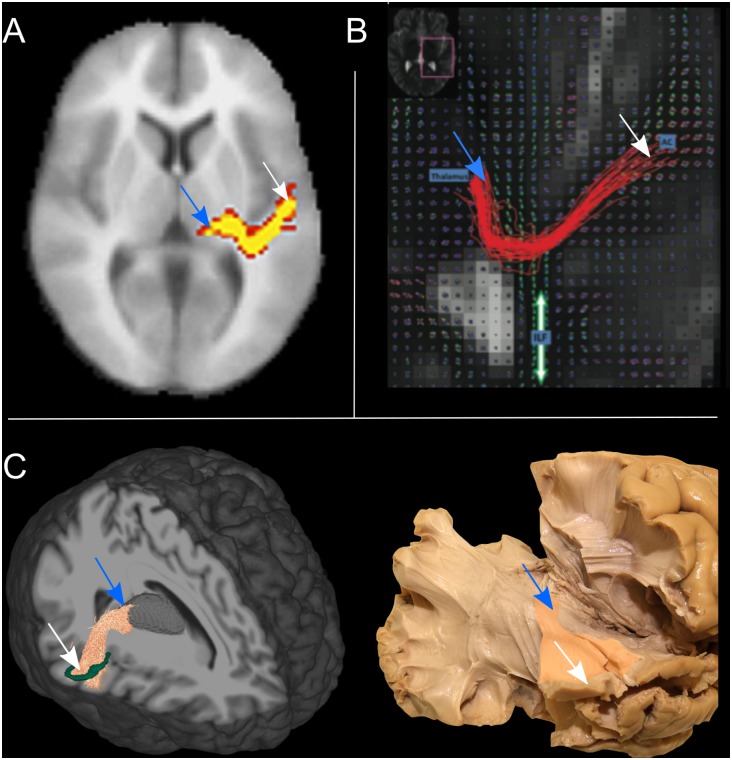
The tractography reconstruction of the acoustic radiation (AR) in three different studies. **(A)** 3D volumetric reconstruction of the right AR in one subject using a multi-tensor model and probabilistic tractography. Voxels are color-coded from 10 (red) to 50 (yellow) samples passing through the voxel (adapted with permission from Behrens et al., 2007). **(B)** The panel shows the streamlines of the right AR in axial view, as reconstructed in one subject using q-ball imaging and probabilistic tractography. The location of the thalamus and auditory cortex (AC) are specified by the blue boxes. The figure also shows the orientation distribution functions (ODF) corresponding to the inferior longitudinal fasciculus (ILF), highlighted by the green arrow (adapted with permissions from Berman et al., 2013). **(C)** Left: 3D tractography reconstruction of the right AR in one subject using constrained spherical deconvolution models and probabilistic tractography. A 3D rendering of the thalamus is also shown in gray and the borders of HG in green. Right: Klinger’s post-mortem blunt dissection of the right AR (modified from Maffei et al., 2018). In all three panels the location of thalamus (blue arrow) and auditory cortex (white arrow) are highlighted.

To address the intrinsic limitations of the tensor formalization, more advanced models have been introduced that can better account for fibers crossing, by modeling more than one fiber population per voxel (Tuch, 2004; Tournier et al., 2008; Descoteaux et al., 2009). These models open up the possibility of propagating streamlines through crossing fiber regions, thus allowing the reconstruction of non-dominant WM bundles, such as the AR. However, together with the low-level diffusion model employed, other parameters play a role in the accurate reconstruction of WM bundles, such as the tractography parameters chosen and the strategy to define inclusion ROIs. Here we report studies that reconstruct the AR 3D tractography profile *in vivo* using multi-fiber-based models (Figure 4), taking into consideration the different combinations of acquisition parameters, diffusion models and ROIs selection strategies that they used (Table 1).

**Table 1:** The table reports the main acquisition and tractography parameters used to reconstruct the AR in the listed studies.

References	Diffusion MRI acquisition parameters	Diffusion model	Inclusion ROIs	Exclusion ROIs	Group size	Success rate
Behrens et al., 2007	60 DWI directions B = 1000 s/mm^2^ 2 × 2 × 2 mm	Ball and stick	MGN (Manual) HG (Single slice)	Other thalamic fibers	9 (HC)	100%
Crippa et al., 2010	60 DWI directions B = 800 s/mm^2^ 1.8 × 1.8 × 2 mm	Ball and stick	Ic (Manual) HG (Manual)	Motor fibers	25 (15 HC, 10 tinnitus)	35–50%
Berman et al., 2013	64 DWI directions B = 3000 s/mm^2^ 2 × 2 × 2 mm	Solid angle q-ball	MGN (Manual) HG (Freesurfer)	Putamen, CC, CG, Pallidum	25 (HC)	98%
Javad et al., 2014	64 DWI directions B = 1400 s/mm^2^ 2.3 × 2.3 × 2.3 mm	Ball and stick	MGN (Manual) HG (fMRI)	Not applied	14 (HC)	71–86%
Profant et al., 2014	64 DWI directions B = 1100 s/mm^2^ 2 × 2 × 2 mm	Ball and stick	Ic (Manual) HG-WM (Freesurfer)	Rostral thalamus, sagittal slice + manual	54 (20 HC, 34 hearing deficit)	100%
Maffei et al., 2018	576 DWI directions B = 1000, 3000, 5000, 10,000 s/mm^2^ 1.5 × 1.5 × 1.5 mm	CSD	TH (FSL) HG (Manual)	Not applied	4 (HC)	100%
Yeh et al., 2018	90 DWI directions B = 1000, 2000, 3000 s/mm^2^ 1.25 × 1.25 × 1.25 mm	QSDR	Clustering + manual labeling	/	842 (HC)	/


*The acquisition parameters column provides the number of diffusion encoding directions, the b-factor (s/mm^2^), and the spatial resolution (mm). The inclusion ROIs column indicates whether seed and target regions were selected manually or with other methods. Some tractography studies performed reconstructions in both directions and results were summed. The success rate column reports the percentage of subjects showing successful AR tractography reconstructions in both hemispheres. DWI, diffusion MRI encoding directions; CG, Cingulate Gyrus; CSD, Constrained Spherical Deconvolution; HG, Heschl’s Gyrus (gray and white matter); HG-WM, Heschl’s Gyrus (only white matter); Ic, Inferior Culliculus; MGN, Medial Geniculate Nucleus; QSDR, q-space diffeomorphic reconstruction; TH, Thalamus; HC, healthy controls.*

Behrens et al. (2007) were able to visualize the course of the AR from the MGN to the cortex using the ball-and-stick model and probabilistic tractography (Behrens et al., 2003) (Figure 4A). The authors demonstrate how this multi-fiber model can reconstruct the AR, overcoming the limitations of the tensor model. Three following studies were then able to demonstrate the reliability of this model for successful *in vivo* reconstruction of the profile of the auditory tract in both healthy subjects and those with tinnitus (Crippa et al., 2010; Javad et al., 2014; Profant et al., 2014). In these studies, different combinations of inclusion ROIs were used to isolate the AR, including the Ic, the MGN, and both functionally and manually defined HG. Nevertheless, the profile of the 3D tractography reconstruction looks visually similar across the four studies and shows connections between the posterior thalamus and the auditory region WM. However, artifacts are visible along the inferior-superior axis in the middle part of the AR at the level of the crossing with the IC and these false positive reconstructions are likely to be related to the probabilistic nature of the diffusion model and tractography algorithm used. Despite the inclusion of false positive signals in the reconstructions, the ball-and-stick model has the important advantage of being accessible to low *b*-value (*b* = 1000 s/mm^2^) diffusion protocols which makes it suitable for clinical investigations of the AR. Berman et al. (2013) (Figure 4B) used a solid-angle q-ball model (Aganj et al., 2010) and probabilistic tractography to successfully reconstruct the auditory connections between the MGN and HG. This reconstruction looks anatomically very accurate and free of false positive artifacts. However, q-ball methods require higher *b*-value diffusion data (b ≥ 3000 s/mm^2^). In these models, the angular resolution of the reconstructed diffusion profiles is increased and the crossing fiber configurations are correctly represented (Tournier et al., 2013), although they pose limitations to its use in clinical populations. Our group (Maffei et al., 2018) used ultra-high *b*-value Human Connectome Project diffusion data-sets (Fan et al., 2015) and spherical deconvolution (Tournier et al., 2008) to reconstruct AR streamlines using probabilistic tractography. We compared the results to Klinger’s post-mortem blunt micro-dissections (Figure 4C), a method based on a brain freezing technique optimized to reveal WM (Ludwig and Klingler, 1956). This approach has been used in several studies to evaluate tractography accuracy (Fernaìndez-Miranda et al., 2015; De Benedictis et al., 2016; Pascalau et al., 2018). The obtained reconstructions agreed with the AR anatomy revealed in the post-mortem dissections and no additional exclusion regions were needed to isolate the AR profile. However, the ultra-high *b*-values used in this study (*b* ≤ 10,000 s/mm^2^) are very rarely achievable, even in research settings. More recently, Yeh et al. (2018) published a population-averaged atlas of several WM connections, including the AR, using q-space diffeomorphic reconstruction (QSDR) (Yeh and Tseng, 2011) and deterministic tractography for multiple fiber orientations. In their approach, the high angular and spatial resolution of the data (1.25 mm isotropic, and *b* ≤ 3000 s/mm^2^) and the large sample (842 subjects) allowed them to reduce the rate of false positive artifacts. However, the AR profile shown in this study, while correctly originating at the posterior thalamus, does not reach the expected auditory cortex on the superior side of the temporal lobe.

Overall, the reconstructed 3D profiles shown in these studies are in accordance with the macrostructural landmarks defined by classic anatomical studies: streamlines originate in the posterior thalamus and course in an antero-lateral direction to terminate in the temporal lobe (Dejerine and Dejerine-Klumpke, 1895). However, AR reconstructions are still highly variable across studies. In particular, while showing similar profiles at the thalamic stemming region, reconstructions differ as they approach the cortex, either falling short of reaching the HG (Crippa et al., 2010; Yeh et al., 2018) or creating false positive artifacts at the intersection with vertically oriented fibers (Javad et al., 2014; Maffei et al., 2018). Moreover, disagreement about the relationship with neighboring tracts exists. Berman et al. (2013) suggest that AR streamlines cross the inferior longitudinal fasciculus (ILF), while Behrens et al. (2007) and Javad et al. (2014) claim that they cross the OR. In a recent work from our group, we did not find that the AR is in close proximity to the ILF (Maffei et al., 2018), supporting older studies that report no crossing between the AR and OR (Pfeifer, 1920; Polyak, 1932), as described above. In addition to variability across studies, low reproducibility across subjects is also reported. Some groups have been able to reconstruct AR tracts on both hemispheres on 100% of subjects (Behrens et al., 2007; Profant et al., 2014; Maffei et al., 2017). In contrast, even when using similar diffusion models (e.g., ball-and-stick), other studies report reconstructions successful in both hemispheres in much lower proportions, such as 35–50% (Crippa et al., 2010) or 71–86% (Javad et al., 2014).

We suggest that the present variability and low reproducibility in the reconstructed AR profile are related to a combination of some of the specific characteristics of the AR that make its tractographic reconstruction quite challenging, even for state of the art tractography techniques: its anatomical location, small size, and inter-individual anatomical variability.

As alluded to in the previous section, the AR constitutes a compact but relatively short and small bundle that lies horizontally in a region with a high density of vertical fibers. Even if multi-fiber models proved capable of representing this crossing, the degree to which this crossing can be accurately resolved in the final 3D reconstruction also depends on the tractography algorithm used and the intrinsic angular resolution of the dMRI data (Tournier et al., 2013). For example, using a higher *b*-value (Berman et al., 2013; Maffei et al., 2018) might help improve accuracy of results relative to those obtained with lower *b*-value data (Crippa et al., 2010). However, in these studies several exclusion ROI have been employed to either constrain tractography (Behrens et al., 2007) or clean the results (Crippa et al., 2010). This renders the accuracy of the final reconstructions sensitive to the selection of these ROI, complicating comparisons across studies.

The use of different ROI selection strategies can strongly affect the resulting tractography reconstructions. The HG is a complex structure that shows large variability in sulcal landmarks across subjects and hemispheres (Rademacher et al., 2001), whereas the MGN is a very small structure, varying from 74 to 183 mm^3^ (Kitajima et al., 2015), making it difficult to locate in neuroimaging data and also highly variable across individuals and hemispheres (Rademacher et al., 2002). Therefore, this variability poses difficulties in the selection of the ROIs used to initiate the tractography reconstruction or to perform the virtual dissections. For example, while reliable automatic segmentation tools for the entire thalamus are available in different public software packages (e.g., FSL^1^, Freesurfer^2^), it is far more challenging to automatically segment smaller structures, such as the MGN, due to both their size and their lower MRI contrast with neighboring WM. Alternative solutions exist but are also challenging. For example, subject-specific manual segmentations of MGN can lead to high anatomical accuracy, but they are very time intensive and, therefore, costly to do in large subject cohorts. Brain atlases also can be used to define the MGN, however given the small size and inter-subject variability, atlas-driven segmentations are unlikely to provide a good anatomical match for all subjects. The development of more accurate automatic parcellation techniques for the thalamic nuclei is expected to improve the accuracy of seed-to-target definition and, thus, of the resulting tractography reconstructions. Recently, a new and promising probabilistic atlas of the thalamic nuclei has been proposed based on a combination of *ex vivo* MRI and histology (Iglesias et al., 2018).

Future research in the field should focus on how to improve the sensitivity and reproducibility of the tractography reconstruction of this bundle. This could be achieved by inputting prior anatomical knowledge in the tractography process, as it is implemented in global-tractography-based frameworks (Yendiki et al., 2011) or more recently developed bundle-specific algorithms (Rheault et al., 2019). Parallel to these advances at the tractography level, there have been efforts in validating tractography results at a micro-anatomical scale. As this validation process progresses, we expect to expand our knowledge of the exact boundaries of the human AR and, consequently, better inform its tractography reconstruction and improve accuracy of tractography results. A more accurate tractography investigation of the AR could expand our structural and functional knowledge of the auditory system, as proposed in the next section.

## The Acoustic Radiation: Functional and Clinical Implications

The reliable *in vivo* reconstruction of the AR in humans may help the exploration of the neuro-anatomical and functional mechanisms underlying auditory processing and language comprehension. The precise characterization of the AR can provide information useful for clinical applications, such as in diagnosis and treatment of hearing and speech disorders, recovery from injury, and performance of interventions that can damage the AR, such as brain surgery or radiation treatments. This section provides a brief review of basic and clinical research areas that could benefit from an improved characterization of the AR.

### Language and Auditory Perception

The ability to communicate through speech is quintessentially human. However, the anatomical organization and the functional mechanisms underlying speech comprehension in the brain are still not understood completely. The acoustic information that reaches the primary auditory cortex via the AR fibers is processed within neural networks that depend on cortico-cortical short- and long-range connections involving temporal, parietal and frontal regions, as schematized in the dual-stream model (Hickok and Poeppel, 2007; Saur et al., 2008; Friederici, 2009). Within this processing network, it is unclear where language-specific processing starts and whether the auditory cortex is involved in speech-specific analysis. Some theories suggest that the left auditory cortex is specialized in processing temporal cues that are fundamental for speech comprehension (Zatorre et al., 2002; Poeppel, 2003) and that this language-specific encoding might actually start at the subcortical level (Hornickel et al., 2009). The diffusion-based reconstruction of the AR, and of the auditory pathways at large, could help address the structural-functional relationship of speech perception. At the structural level, it would be interesting to understand whether the AR exhibits a degree of leftward lateralization in its volume, as demonstrated for some of the other WM bundles implicated in language processing (Catani et al., 2007). Reports on the macroscopic volumetric asymmetry of cortical auditory regions have been known for some time (Von Economo and Horn, 1930; Galaburda et al., 1978; Geschwind and Galaburda, 1985; Penhune et al., 1996), but only one study specifically investigated the hemispheric lateralization of the AR (Bürgel et al., 2006). At the functional level, the recent association of tractography and neurophysiological techniques [such as magnetoencephalography (MEG) and electroencephalography (EEG)] opens interesting possibilities for investigating these topics. EEG metrics have been recently correlated with diffusion metrics in the investigation of the OR (Renauld et al., 2016). Similarly, EEG/MEG- and tractography-derived measures could be combined to investigate the relationship between temporal cortical regions and auditory function in both healthy subjects and patients.

On a finer scale, AR streamline terminations could be combined with functional MRI to provide critical insights into the subdivision of the auditory cortex, the borders of which are still not clearly defined using *in vivo* neuroimaging methods (Baumann et al., 2013). In this sense tractography could be used to investigate the topographical organization of the AR with respect to the different subdivisions of the auditory cortex. The different auditory cortical regions show a hierarchical organization in information processing (Tardif and Clarke, 2001), from highly specialized core regions to more integrated tertiary para-belt regions. This is confirmed by functional MRI studies, which suggest a gradient of increasingly more complex and abstracted processing from primary to higher-order auditory regions (Rauschecker et al., 1995; Humphries et al., 2014). Direct connections from MGN to secondary regions have been shown in monkeys (Rauschecker et al., 1997), suggesting that this functional organization might be maintained in the topographical organization of the AR fibers. At its present stage, tractography can locate and delineate the profile of major WM bundles with some accuracy, but it is very difficult to achieve precise site-to-site connectivity analysis with it; this limits the *in vivo* investigation of the WM topographical organization of the human brain. However, some studies use diffusion tractography to parcellate functionally different cortical regions (Rushworth et al., 2006; Anwander et al., 2007) and investigate the topographical organization of major bundles (Lee et al., 2016), and new methods have been proposed to advance the use of tractography for this purpose (Aydogan and Shi, 2016). In this scenario, it would be interesting to investigate whether some language-specific connections exist inside the AR and whether these project to higher-order language-specific cortical regions. Moreover, this would also allow for the investigation of whether the tonotopical organization of the primary auditory cortex is reflected in its thalamo-cortical connections, as was recently shown in the mouse brain (Hackett et al., 2011). As dMRI acquisition (in particular, spatial resolution), diffusion modeling and tractography techniques improve, we will be able to bridge the gap between the micro-anatomical knowledge we have of the thalamo-cortical connections in animals and the macro-anatomical description in humans.

#### Language and Hearing Disorders

Damage to the auditory regions, most often the result of brain infarct or traumatic injury, has been associated generally with rare auditory syndromes, such as verbal auditory agnosia (Shivashankar et al., 2001), environmental auditory agnosia (Taniwaki et al., 2000), and cerebral (or central) deafness (Griffiths, 2002). However, until now, only one study investigated the extent of WM damage to the AR in a patient suffering from verbal auditory agnosia (Maffei et al., 2017). Investigating the extent of damage to the AR in patients suffering speech-related comprehension deficits would potentially enhance our understanding of the involvement of the AR in language processing.

Also, there is evidence that AR infarct can cause auditory hallucination (Woo et al., 2014), and that the extra-lemniscal pathway might be implicated in tinnitus perception (Moller et al., 1992). At present, studies investigating the auditory pathways in these patients relied on WM ROI measurements (Lee et al., 2007; Lin et al., 2008), which only outline a portion of the underlying WM bundles and may not be representative of the entire tract. Being able to better understand the dynamics and location of such changes in the auditory pathways could help inform pathophysiological treatment strategies, such as repetitive transcranial stimulation (Langguth et al., 2010).

In congenitally and early deaf subjects, volumetric studies have outlined differences in gray and white matter of the auditory regions, compared to hearing subjects (Shibata, 2007; Kim et al., 2009), but to the best of our knowledge, no specific study on the AR in deaf subjects has been conducted to date. In addition to providing more detailed information on the anatomical changes occurring in the brain as a consequence of sensory deprivation, tractography of the AR may serve as an additional early diagnostic as well as complementary treatment tool in monitoring data in congenital hearing loss. This would help avoid delayed diagnosis that might lead to poor speech outcomes (Dedhia et al., 2018). Moreover, AR tractography reconstruction might be fundamental in assessing auditory pathway integrity before and after cochlear implantation, potentially predicting implant success (Huang et al., 2015).

The structural-functional relationship in language and hearing disorders can be further investigated by combining tractography reconstructions and more recently developed diffusion measures (Raffelt et al., 2012; Calamuneri et al., 2018) within both classical and more advanced tractography frameworks (Daducci et al., 2016). The application of these methods in clinical populations in the context of hearing disorders may help characterize axonal and myelination diseases (Ohno and Ikenaka, 2018) and auditory neuropathies (Moser and Starr, 2016; Ohno and Ikenaka, 2018).

Tractography reconstruction of the AR could help us investigate the anatomy of this tract in patients with hearing and/or language disorders, understand whether these fibers undergo structural reorganization in the case of auditory deprivation, and clarify the extent of AR damage in post-stroke lesion profiles. This may be critical for shedding light on the functional-structural relationships of linguistic and non-linguistic sound processing in the human brain.

#### Brain Surgical Planning

Investigating the functional and anatomical characteristics of the auditory fibers reaching the cortex, especially in relation to their implications for language function, would be important for surgical planning, such as in the case of tumor or epilepsy surgery (Wu et al., 2007; Farshidfar et al., 2014). The 3D reconstruction of major WM bundles is employed to plan and guide resections during surgery, and a functional atlas of human WM to drive well balanced onco-functional resections has been proposed recently (Sarubbo et al., 2015). In this context, diffusion-based virtual dissections have focused almost exclusively on language and sensory-motor structures (Chen et al., 2015). Possible reasons why AR fibers have not received much attention in the neurosurgical literature include the possibility that most of the non-linguistic auditory processing may happen at the brain-stem level and that auditory information is conveyed to both hemispheres, so that extensive bilateral damage is necessary for complete deafness (Griffiths, 2002). However, different sub-modal aspects of auditory processing, for example, those related to music perception and/or speech comprehension, might depend on the integrity of these projections (Hayashi and Hayashi, 2007; Baird et al., 2014). For cases of temporal lobe resection, the reliable virtual reconstruction of the AR might be critical for minimizing post-operative deficits in these domains. Also, it might serve in pre-operative assessments for cochlear implantation, as hearing recovery after implantation is influenced by the integrity of subcortical pathways (Vlastarakos et al., 2010).

### Brain Radiation Oncology Planning

Today, X-ray therapy (XRT) is the standard of care for most brain tumors. However, XRT can damage normal brain tissue, causing neurocognitive deficits in different cognitive domains (Makale et al., 2016). The consideration of neuroimaging techniques for treatment planning is gaining importance as it helps avoid such complications by minimizing the unnecessary absorption of radiation in sensitive regions outside the tumor. Nevertheless, further improvement of radiation treatment will require tailored radiotherapy based on intra-treatment response (Wong et al., 2017).

Additionally, studies have shown XRT side effects in both gray (Bahrami et al., 2017) and white matter, although it is still largely unclear how variable sensitivity to radiation injury is across various regions of the brain (Connor et al., 2017). Kawasaki et al. (2017) show that tractography can help evaluate how radiation from XRT differentially affects WM regions and pathways. This knowledge, together with an understanding of how damage to such regions and pathways affects cognitive processes, could be used in the future to further optimize radiation treatment planning.

## Conclusion

The anatomical and functional organization of the auditory system is still not well understood, particularly in humans. Successful *in vivo* tractographic reconstruction of the human auditory tracts is of great importance for clinical applications (e.g., pre-surgical mapping), as well as for basic research (e.g., language and auditory systems). This review outlines how the characterization of the AR has been limited by the methods used in the past and how advances in MRI acquisition and diffusion tractography methods offer the possibility to improve the characterization of this important WM tract. A few exciting potential research areas are suggested that would investigate anatomy and function concurrently in the same individual, both in health (e.g., the role of these tracts in language processing) and in disease (e.g., how the integrity of this tract relates to cognitive deficits). However, in order to obtain reliable reconstructions of the AR across subjects and protocols, additional work is needed to better understand how diffusion MRI acquisition and tractography reconstruction strategies affect the AR 3D characterization and to validate tractography reconstructions at a micro-anatomical scale. Furthermore, as diffusion tractography is blind to the directionality of reconstructed fibers, the AR bundle could include both thalamo-cortical and cortico-thalamic projections, and therefore more studies are needed to differentiate between the afferent or efferent nature of these connections. Although these methodological challenges apply to diffusion MRI tractography in general, here we have focused on their relevance to the AR, a tract that has proven to be rather elusive for the reasons herein reviewed.

## Author Contributions

JJ conceived the review. SS revised the manuscript for neuro-anatomical content. CM drafted the manuscript and designed the figures. All authors contributed to the final manuscript.

## Conflict of Interest Statement

The authors declare that the research was conducted in the absence of any commercial or financial relationships that could be construed as a potential conflict of interest.

## Footnotes

1 https://fsl.fmrib.ox.ac.uk/fsl/

2 http://surfer.nmr.mgh.harvard.edu/

## Abbreviations

AC: auditory cortex
AR: acoustic radiation
CC: corpus callosum
CR: corona radiata
CSD: constrained spherical deconvolution
dMRI: diffusion magnetic resonance imaging
DWI: diffusion weighted imaging
EC: external capsule
EEG: electroencephalography
FA: fractional anisotropy
FS: sylvian fissure
HC: healthy cotrols
HG: Heschl’s gyrus
Ic: inferior culliculus
IC: internal capsule
ICPL: posterior limb of the internal capsule
ICSL: sub-lenticular part of the internal capsule
ILF: inferior longitudinal fasciculus
LGN: lateral geniculate nucleus
MEG: magnetoencephalography
MGN: medial geniculate nucleus
ODF: orientation distribution function
OR: optic radiation
ROI: region of interest
PAC: primary auditory cortex
SNR: signal-to-noise ratio
STG: superior temporal gyrus
STP: superior temporal plane
TH: thalamus
WM: white matter
XRT: X-ray therapy
